# No longitudinal association between hearing loss and Alzheimer’s disease pathology

**DOI:** 10.1016/j.tjpad.2026.100481

**Published:** 2026-01-20

**Authors:** Jordi H.C. Boons, Phuong Thuy Nguyen Ho, Anna van Houwelingen, M. Arfan Ikram, Gertjan Dingemanse, Bernd Kremer, Meike W. Vernooij, Andre Goedegebure, Julia Neitzel

**Affiliations:** a Department of Otorhinolaryngology, Head & Neck surgery, Erasmus MC, University Medical Center Rotterdam, Dr. Molewaterplein 40, 3015 GD Rotterdam, the Netherlands; b Department of Radiology and Nuclear Medicine, Erasmus MC, University Medical Center Rotterdam, Dr. Molewaterplein 40, 3015 GD Rotterdam, the Netherlands; c Department of Epidemiology, Erasmus MC, University Medical Center Rotterdam, Dr. Molewaterplein 40, 3015 GD Rotterdam, the Netherlands

**Keywords:** Amyloid PET, Plasma biomarkers, Hearing loss, Longitudinal, Alzheimer’s disease, Risk factor

## Abstract

**Introduction:**

Hearing loss (HL) is a potential risk factor for Alzheimer’s disease (AD), with animal studies suggesting a bidirectional relationship. This study examines whether HL links to changes in AD pathology and, whether AD biomarkers relate to subsequent HL progression.

**Methods:**

Baseline Aβ42/Aβ40 and p-tau217 were measured using single-molecule arrays in 474 participants of the Rotterdam Study (mean age=62.37) between 2010-2016. HL was defined as the better-ear’s pure-tone threshold average. After seven years, participants underwent amyloid PET; HL and p-tau217 were reassessed two years after PET.

**Results:**

Baseline HL was not associated with amyloid PET positivity, Aβ42/Aβ40, or longitudinally with p-tau217. Likewise, HL progression did not predict PET outcomes. APOE4 carriership did not modify associations with Aβ PET. Similarly, baseline plasma biomarkers were also unrelated to longitudinal HL changes.

**Conclusion:**

No bidirectional association was observed between HL and AD pathology, suggesting that HL may contribute to dementia through other pathways.

## Highlights

• Baseline hearing loss (HL) was not linked to longitudinal changes in p-tau217.
• Baseline HL was not associated with incident amyloid PET positivity.
• HL progression did not predict PET outcomes.
• APOE4 carriership did not modify associations with Aβ PET.
• Baseline plasma biomarkers were also unrelated to longitudinal HL changes.

## 1 Introduction

Dementia is recognized by the World Health Organization as one of the major causes of disability and care dependence among older people worldwide [1]. This condition poses a significant global economic burden due to its increasing prevalence [2]. Therefore, identifying and mitigating risk factors for dementia has become a global health priority. Previous research has linked hearing loss (HL) to an increased risk of developing dementia as well as Alzheimer’s disease (AD) [[3], [4], [5]]. Building on this evidence, the 2024 Lancet Commission identified HL as one of the modifiable risk factors for dementia, which is suggested to be particularly relevant during midlife [3]. One proposed mechanism linking HL and dementia is the sensory deprivation theory. This hypothesis states that prolonged sensory deprivation from HL may induce pathological changes in brain tissue, contributing to cognitive decline [4]. However, whether HL is directly linked to core AD neuropathological hallmarks remains unclear.

AD pathology can begin decades before clinical symptoms appear, starting with the aggregation of amyloid-β (Aβ) plaques, which then drive the accumulation of tau neurofibrillary tangles. Early AD pathology manifests as a decrease in the long-to-short Aβ oligomers ratio (Aβ42/Aβ40) in blood plasma [[6], [7], [8]]. This decrease indicates the withdrawal of Aβ42 from the blood and its accumulation into Aβ plaques within the brain. These plaques can be detected by positron emission tomography (PET), which uses a radioactive tracer that binds to cortical Aβ plaques. A more recently developed blood-based biomarker is phosphorylated mid-region Tau at codon 217 (p-tau217). Plasma p-tau217 measures Aβ-mediated hyperphosphorylation of tau proteins in soluble form, reflecting a mix of Aβ- and tau-related signals [6,9]. Together, plasma Aβ42/Aβ40, p-tau217, and Aβ PET provide a comprehensive assessment of early AD pathology [10,11].

Findings from human studies on the relationship between HL and AD biomarkers remain inconclusive [12]. Most studies have focused on HL in late life [[13], [14], [15], [16], [17], [18], [19], [20]], with only a few examining this relationship in midlife [[21], [22], [23], [24]]. Golub et al. (2021) and Irace et al. (2022) reported an association between larger hearing losses and higher Aβ PET pathology, using data from the Northern Manhattan Study of Metabolism and Mind (NOMEM) study [23,24]. In contrast, Wang et al. (2022) found no relationship between hearing impairment and cerebrospinal fluid (CSF) Aβ42 in participants of the Chinese Alzheimer’s Biomarker and Lifestyle (CABLE) and Alzheimer’s Disease Neuroimaging Initiative (ADNI) studies [21]. However, a link was observed with CSF pTau181 in the CABLE cohort. Notably, these studies possess limitations that may restrict their generalizability: Golub et al. [23] and Irace et al. [24] utilized small samples in their analysis (n=98), while the CABLE cohort relied on self-reported hearing impairment [21].

Notably, an animal study has proposed a pathway through which HL could exacerbate AD pathology by inhibiting the growth/differentiation factor 1 pathway [25]. In addition, other studies have shown that mice with AD had poorer hearing compared to controls [26,27]_._ These findings suggest a potential bidirectional relationship between HL and AD, although this has been difficult to establish in human studies. Recent studies increasingly support the hypothesis that HL contributes to dementia risk, including AD [5,28]. However, establishing the reverse direction is challenging in human research, yet it remains an important avenue for investigation when study design permits a bidirectional assessment. Findings from animal studies and the restricted generalizability from human studies highlight the urgent need for further research into the potential bidirectional relationship between HL and AD biomarkers, with sufficient sample size, objective measurement of HL, and a focus on an age range more susceptible to HL onset.

In this study, we aimed to investigate the potential bidirectional relationship between HL and AD biomarkers in mid to late life in 474 cognitively unimpaired participants from the population-based Rotterdam Study. We assessed hearing function and collected AD plasma biomarkers at baseline and after a nine-year follow-up. In addition, participants underwent Aβ PET imaging at seven years post-baseline. We performed the following analyses. First, we tested whether HL relates to subsequent AD pathology by examining associations between baseline hearing function and (1) longitudinal changes in plasma p-tau217 levels and (2) incident Aβ PET positivity at follow-up. Lastly, (3) we tested the reverse direction, examining whether baseline AD biomarkers (p-tau217 and Aβ42/Aβ40 levels) relate to longitudinal hearing decline.

## 2 Methods

### 2.1 Setting

The current study utilized data from the prospective population-based Rotterdam Study, which is located in the district of Ommoord, Rotterdam, the Netherlands [[29], [30], [31]]. The Rotterdam Study consists of four subcohorts: beginning in 1990, the first cohort (RS-I) started with 7,983 participants aged 55+; the second cohort (RS-II) was initiated in 2000 with 3,011 participants [32]. In 2006, the third cohort (RS-III) began with 3,932 participants (aged 45+). Every three to six years, participants are interviewed at home and then undergo an extensive examination at the research center. From 2005, participants also underwent brain magnetic resonance imaging (MRI) based on a standard imaging protocol [29]. Hearing assessment was added to the study protocol from 2011 onwards [29]. The Rotterdam Study was approved by the Medical Ethics Committee of Erasmus MC. Written informed consent was obtained from all participants.

**Fig. 1:**
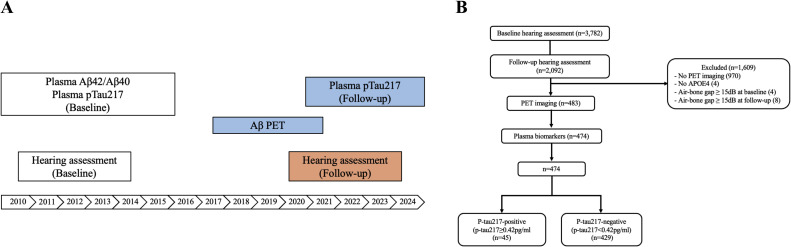
(A) Study design, (B) Participant selection.

Between 2011 and 2014, a total of 3,782 participants from the RS-II and RS-III cohort had an audiometric assessment, of which 2,092 (55.3%) had a follow-up measurement in 2021-2024. From 2018 to 2021, a subsample of the RS-II and RS-III had PET imaging. We included participants that met the following criteria: (1) at least 60 years old at the time of the PET; (2) had a valid brain MRI between 2011 and 2016 (required for brain image alignment); (3) no PET contraindications; (4) no dementia diagnosis [33]. In total, 639 participants underwent PET imaging. Of these participants, 628 had plasma Aβ42/Aβ40 and p-tau217 collected between 2010 and 2016, and 572 had plasma p-tau217 collected between 2021 and 2024; 474 participants had all relevant measures. We excluded participants with an air-bone gap in the hearing assessment equal to or above 15 dB at baseline (n = 4) and follow-up (n = 8), as these may reflect temporary HL because of the conductive origin. We defined the air-bone gap as the mean difference between the air-conduction hearing thresholds and bone-conduction thresholds at 500 and 4000 Hz. Overall, we included participants if they had a valid PET scan, APOE4 data, and HL data at baseline and follow-up (n=474). Fig. 1 summarizes the study design (A) and participant selection (B).

### 2.2 Pure-tone audiometry

Hearing thresholds in decibels (dB) hearing level were determined with pure-tone audiometry in a soundproof booth. A computer-based audiometry system (Decos Technology Group, version 210.2.6, AudioNigma interface) and TDH-39 headphones were used. Hearing thresholds were measured according to ISO standard 8253-1 [34]. Air conduction (0.25–8 kHz) and bone conduction (0.5 and 4 kHz) thresholds were assessed for both ears, with masking applied when appropriate, using the Hood method [35]. We considered the ear with the lowest average hearing thresholds across all frequencies to be the best hearing ear. The better hearing ear was chosen as it best represents daily life function and avoids the inclusion of possible (temporary) incidental HL at the worse hearing ear, unrelated to aging. When the average hearing thresholds were equal, the left or right ear was chosen alternately. HL was defined as the pure-tone threshold average in dB of the speech frequencies (0.5 – 4 kHz) of the better hearing ear. We calculated the annual change in HL by determining the difference in HL between the follow-up and baseline measurements for the better hearing ear at baseline, as defined above, and dividing by the follow-up time. The degree of HL of the better hearing ear was also classified to characterize the study population, following the WHO Grades of hearing impairment [36].

### 2.3 AD plasma biomarkers

Ethylenediaminetetraacetic acid (EDTA) plasma was collected, aliquoted, and stored at −80°C. Plasma analyses were performed at the Neurochemistry Laboratory, Amsterdam UMC, VUmc, the Netherlands, using a Simoa HDx analyzer (Quanterix, Billerica, MA, USA). Plasma p-tau217 was measured using the Simoa® p-tau217 Alzpath CARe Advantage Kit at baseline and the Simoa® p-tau217 Alzpath Advanced Plus Kit at follow-up. To ensure comparability between timepoints, 15 baseline samples were measured with both assays to create a bridging formula that harmonized values across kits. The correlation between duplicate measurements was excellent (Spearman's ρ = 0.971), confirming strong assay concordance. Plasma Aβ40 and Aβ42 were assessed at baseline using the Simoa® Neuro 4-Plex E Kit. We excluded data if the concentration was outside the assays’ lower and upper limits of quantification (n = 2).

### 2.4 Amyloid positron emission tomography

18F-forbetaben amyloid PET imaging was performed and processed following an established pipeline [33]. We acquired PET scans 90-110 minutes after an intravenous injection of 300MBq ^18^F-florbetaben (Neuraceq, Life Molecular Imaging GmbH) and recorded for 20 minutes in listmode using a Siemens Biograph mCT PET/CT (Siemens Healthineers). Scatter and attenuation corrections were applied using a low-dose CT (120 kVp and 40 quality reference mAs) acquired before PET imaging.

Structural T1-weighted images were acquired using a Signa Excite II GE 1.5T scanner (GE Healthcare) and processed with Freesurfer (v5.1.0). Parcellation was performed using the Desikan-Killiany atlas [37]. Low-dose CT scans and co-registered PET images were aligned to the T1-weighted structural images in SPM (v12) using rigid body registration, followed by a visual quality assessment. Freesurfer-based parcellation from each participant’s high-resolution structural T1 image was then applied to the PET image.

We then quantified the level of beta-amyloid plaque deposition in the brain using the average cortical standardized uptake value ratio (SUVR). It was calculated by dividing the mean amyloid PET tracer uptake across five cortical regions (frontal, cingulate, lateral parietal, and lateral temporal cortices) by the mean tracer uptake in the cerebellum reference region [38,39]. Additionally, we determined the SUVR of the left and right superior temporal gyri (segmented by Freesurfer), which contain the primary auditory cortex [40].

Amyloid-beta status (negative/positive) was determined using a hybrid algorithm that combined quantitative SUVR thresholds with qualitative visual assessment [41]. An SUVR > 1.24 was deemed positive and < 1.10 as negative [42]. An SUVR between 1.10 and 1.24 was deemed positive only if the visual read was labelled positive by two independent raters.

Incident PET Aβ positivity was defined as progressing from a negative p-tau217 level (< 0.42pg/mL [9]) at baseline to a positive Aβ PET scan at follow-up.

### 2.5 Covariables

In this study, we controlled for age (years), sex (female/male), education (primary, lower, intermediate, higher), time between predictor and outcome, estimated glomerular filtration rate (eGFR, for analysis with plasma biomarkers), and baseline HL (for analysis with changes in HL) (model 1). In a second model, we additionally controlled for APOE4 carriership (yes/no) (model 2). In a third model, we additionally controlled for body mass index (BMI, kg/m^2^), hypertension (yes/no), diabetes(yes/no), smoking (never/past/current), and alcohol consumption (g/day) (model 3).

Education was defined based on the UNESCO classification (1=primary education (primary), 2=lower/intermediate general education or lower vocational (low), 3=intermediate vocational education or higher education (intermediate), 4=higher vocational education or university (high) [43,44]. During home visits, information about education, smoking, and alcohol consumption was collected. BMI, hypertension, diabetes, and eGFR were measured at the research center. Hypertension was defined as systolic blood pressure >140 mmHg, diastolic blood pressure >90 mmHg, or the use of blood pressure-lowering medication. Diabetes was defined as fasting serum glucose ≥7.0 mmol/l, non-fasting serum glucose levels ≥11.0 mmol/l, or the use of blood-glucose-lowering medication. eGFR was estimated using serum creatinine.

### 2.6 Statistical analysis

All analyses were performed using R version 4.3.2. Descriptive statistics comparing baseline p-tau217-negative and p-tau217-positive participants were assessed using t-tests for continuous variables and chi-square tests for categorical variables. Multiple imputation with 5 iterations was used to handle missing covariates, which were limited to 2 missing values for education level and diabetes status, and 14 missing values for eGFR at follow-up. Separate imputation was performed for models 1, 2, and 3, which included all covariates used in each statistical model. All continuous variables were scaled before analysis. All reported coefficients were standardized estimates.

For aim 1, testing HL as a risk factor for AD pathology, we used linear mixed effects models with random intercepts for participants to determine the association between HL at baseline and changes in plasma p-tau217 over time (HL x time interaction). P-tau217 was log-transformed to meet the assumptions for linear mixed models.

Complementary to this analysis, we applied logistic regression to determine whether baseline HL is associated with Aβ PET positivity in the whole study population. To minimize reverse-causation, we performed a secondary analysis in which we excluded participants with baseline p-tau217 levels ≥ 0.42 pg/mL (n = 45), as these individuals are likely to already have Aβ pathology at baseline [9]. This approach allowed us to examine whether baseline hearing loss was associated with newly emerging Aβ accumulation over the follow-up period. Similarly, we investigated the association between change in HL and incident Aβ PET positivity, adjusting for baseline HL. This was also performed in the whole sample and baseline p-tau217-negative subgroup.

Sensitivity analyses were performed using continuous PET SUVR as the outcome instead of binary PET positivity to investigate whether the binarization resulted in a possible bias. We used linear regression models for these sensitivity analyses. After fitting the models, heteroscedasticity was identified. To account for this, robust standard errors were computed using the *sandwich* package. Additional linear regression analyses were performed using the SUVR of the superior temporal gyri to elucidate whether the association between Aβ pathology and HL may be more apparent in the auditory cortex. We further investigated whether APOE4 carriership modified the relationship between HL and PET SUVR by including a multiplicative interaction term (HL*APOE4 carriership). All sensitivity analyses were performed with both the whole sample and baseline p-tau217-negative subgroup.

For aim 2, testing AD pathology as a determinant factor of subsequent HL, we applied linear mixed effects models with random intercepts for participants to examine whether AD plasma biomarker (p-tau217 and Aβ42/Aβ40) levels at baseline predicted the rate of change in HL. Each model included baseline biomarker level, time, and the biomarker x time interaction term.

## 3 Results

### 3.1 Sample characteristics

This study included 474 participants with a mean age at baseline of 62.4 (SD = 5.2) years. The sample included 51.3% women and 34.9% completed higher education. Approximately 31.2% of the participants were APOE4 carriers. Baseline HL was on average 17.2 dB (normal hearing is between 0 – 25 dB), where 15.8% of the participants had hearing impairment (HL > 25 dB). Abnormal p-tau217 levels (≥ 0.42 pg/mL) were observed in 45 participants at baseline. Approximately seven years after the baseline visit, Aβ PET was performed. 15.8% of the participants were Aβ PET positive. About two years after PET, HL and plasma p-tau217 were collected a second time. As expected, both average HL (25.3 dB), the percentage of participants with hearing impairment (42.0%), and p-tau217 levels (0.39 pg/mL) were elevated at follow-up relative to baseline. Table 1 shows all sample characteristics.

**Table 1:** Sample characteristics.

Variables	Levels	All participants	Baseline p-tau217-positive	Baseline p-tau217-negative	p
n		474	45	429	
**Demographics**					
Age at baseline, mean (SD)		62.37 (5.22)	66.13 (6.88)	61.98 (4.86)	<0.001
Sex (%)	Male	231 (48.7)	37 (82.2)	194 (45.2)	<0.001
	Female	243 (51.3)	8 (17.8)	235 (54.8)	
Education (%)	Primary	29 (6.1)	3 (6.7)	26 (6.1)	0.453
	Lower	131 (27.7)	8 (17.8)	123 (28.7)	
	Intermediate	148 (31.3)	15 (33.3)	133 (31.1)	
	Higher	165 (34.9)	19 (42.2)	146 (34.1)	
**Amyloid biomarkers**					
SUVR, mean (SD)		1.04 (0.17)	1.24 (0.29)	1.02 (0.13)	<0.001
Aβ status (%)	Negative	399 (84.2)	21 (46.7)	378 (88.1)	<0.001
	Positive	75 (15.8)	24 (53.3)	51 (11.9)	
Plasma Aβ42/Aβ40 ratio, mean (SD)		0.06 (0.01)	0.06 (0.01)	0.06 (0.01)	0.001
Baseline plasma p-tau217 (pg/mL), mean (SD)		0.27 (0.14)	0.59 (0.20)	0.23 (0.07)	<0.001
Follow-up plasma p-tau217 (pg/mL), mean (SD)		0.39 (0.29)	0.91 (0.52)	0.33 (0.19)	<0.001
**Pure-tone average**					
Baseline HL (dB), mean (SD)		17.2 (9.2)	18.7 (7.9)	17.1 (9.4)	0.265
Degree of HL at baseline					
	No impairment (≤25 dB)	399 (84.2)	36 (80.0)	363 (84.6)	0.422
	Slight impairment (26-40 dB)	64 (13.5)	9 (20.0)	55 (12.8)	
	Moderate impairment (41-60 dB)	10 (2.1)	0 (0.0)	10 (2.3)	
	Severe impairment (61-80 dB)	1 (0.2)	0 (0.0)	1 (0.2)	
Follow-up HL, mean (SD)		25.3 (11.8)	27.1 (10.9)	25.1 (11.9)	0.279
Degree of HL at follow-up					
	No impairment (≤25 dB)	275 (58.0)	21 (46.7)	254 (59.2)	0.347
	Slight impairment (26-40 dB)	141 (29.7)	18 (40.0)	123 (28.7)	
	Moderate impairment (41-60 dB)	55 (11.6)	6 (13.3)	49 (11.4)	
	Severe impairment (61-80 dB)	3 (0.6)	0 (0.0)	3 (0.7)	
Yearly HL change, mean (SD)		0.9 (0.6)	0.9 (0.7)	0.90 (0.6)	0.678
**Other covariates**					
Time plasma biomarkers and HL at follow-up, mean (SD)		8.96 (0.90)	8.98 (1.12)	8.96 (0.88)	0.915
Time between baseline HL and PET, mean (SD)		6.96 (1.11)	7.16 (1.43)	6.94 (1.07)	0.223
Time between follow-up HL and PET, mean (SD)		2.00 (0.67)	1.82 (0.65)	2.02 (0.67)	0.060
APOE4 carriership (%)	No	326 (68.8)	27 (60.0)	299 (69.7)	0.243
	Yes	148 (31.2)	18 (40.0)	130 (30.3)	
eGFR (µmol/L), mean (SD)		85.08 (13.69)	75.95 (19.65)	86.04 (12.55)	<0.001
BMI, mean (SD)		27.12 (3.88)	26.72 (3.34)	27.16 (3.94)	0.467
Smoking (%)	Never	145 (30.6)	11 (24.4)	134 (31.2)	0.577
	Past	241 (50.8)	26 (57.8)	215 (50.1)	
	Current	88 (18.6)	8 (17.8)	80 (18.6)	
Hypertension (%)	No	213 (44.9)	17 (37.8)	196 (45.7)	0.391
	Yes	261 (55.1)	28 (62.2)	233 (54.3)	
Diabetes (%)	No	433 (91.5)	34 (77.3)	399 (93.0)	0.001
	Yes	40 (8.5)	10 (22.7)	30 (7.0)	
Alcohol (gr/day), mean (SD)		8.57 (8.94)	10.71 (9.60)	8.35 (8.85)	0.091

Abbreviations: SD, standard deviation; APOE4, Apolipoprotein E4; SUVR, standard uptake value ratio; Aβ42/Aβ40, amyloid-beta 42 to amyloid-beta 40 ratio; Aβ, amyloid-beta; p-tau217, phosphorylated tau at threonine 217; HL, hearing loss; BMI, body mass index; eGFR, estimated glomerular filtration rate.

Missing: follow-up plasma p-tau217 (n = 1), education (n = 2), diabetes (n = 2).

### 3.2 Hearing loss at baseline and subsequent changes in plasma p-tau217 levels

**Fig. 2:**
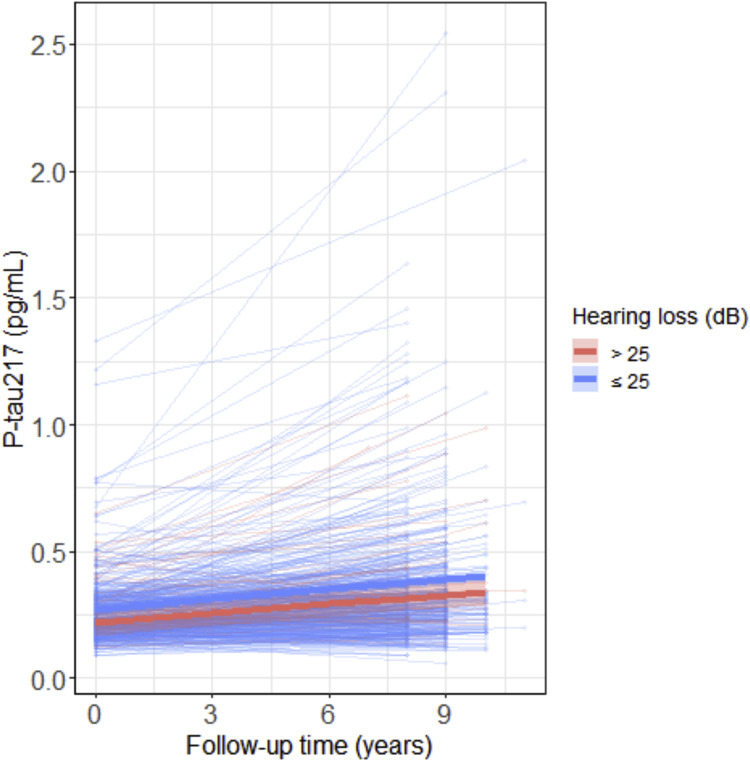
Scatterplots showing the association between changes in p-tau217 levels over time and hearing loss at baseline (HL: normal hearing between 0 – 25 dB vs. impaired hearing > 25dB). The shaded area indicates the 95% CI around the mean derived from a linear mixed effects model. Note that the classification into normal vs impaired hearing was performed for visualization purposes only.

We found no statistically significant relationship between baseline HL and baseline p-tau217 levels (model 1: β=0.00 [-0.08, 0.08], *p*=1.00; model 2: β=0.00 [-0.08, 0.08], *p*=0.98; model 3: β=0.00 [-0.07, 0.08], *p*=0.93) or between baseline HL and longitudinal changes in p-tau217 over time (model 1: β=0.02 [-0.01, 0.05], *p*=0.27; model 2: β=0.02 [-0.01, 0.05], *p*=0.27; model 3: β=0.02 [-0.01, 0.05], *p*=0.26). When expressed in original units, each 10 dB increase in HL (reflecting one level of greater severity) was associated with a 0.01 pg/mL decrease in plasma p-tau217 levels at baseline and an annual increase of 0.001 pg/mL in plasma p-tau217, although neither association reached statistical significance. Taken together, this indicates that baseline HL is not associated with cross-sectional levels or longitudinal changes in plasma p-tau217 levels (**Table S1**). Fig. 2 shows the individual p-tau217 trajectories across the nine-year follow-up period for the group with normal hearing versus impaired hearing (HL > 25dB) at baseline.

### 3.3 Hearing loss at baseline and incident Aβ PET positivity

**Fig. 3:**
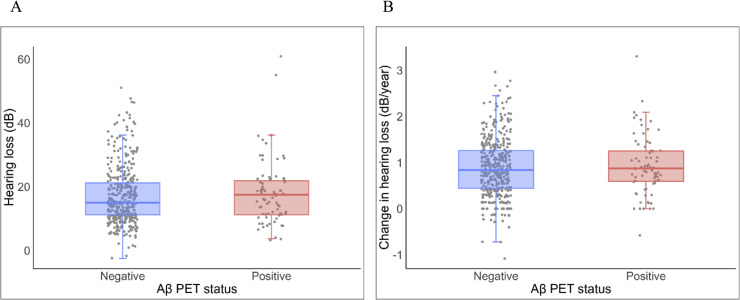
Boxplots of **A** HL at baseline and **B** yearly change in HL for participants who are Aβ-negative (blue) and Aβ-positive (red) on PET. Each point represents a single participant.

Fig. 3A shows boxplots of HL baseline values for Aβ PET-positive and -negative participants. No significant difference was found between the two groups. In logistic regression models, HL was not a significant predictor of *incident* Aβ PET positivity seven years later (Table 2). In a sensitivity analysis, the linear relationship between HL and Aβ PET SUVR was not significant (**Table S2; Fig. S1A**). Associations were also non-significant for the SUVR in both the left and right superior temporal gyri (**Table S2; Fig. S1B-C**). All of the aforementioned results were similar for the total study population and the baseline p-tau217 negative participants (Table 2**; Table S2**). Furthermore, we found no interaction between APOE4 carriership and HL on PET SUVR (β=0.10 [-0.11, 0.31], *p*=0.37; **Fig. S1D**).

**Table 2:** Logistic regression results of the association between hearing loss or change in hearing loss and Aβ PET positivity.

		OR	95%CI	*p*	OR	95%CI	*p*
		*All participants (N = 474)*			*Baseline p-tau217-negative participants (n = 429)*		
Baseline HL	Model 1	0.98	0.75, 1.28	0.88	1.05	0.77, 1.43	0.78
	Model 2	0.94	0.71, 1.24	0.66	0.99	0.72, 1.35	0.93
	Model 3	0.95	0.72, 1.27	0.74	0.98	0.71, 1.36	0.91
HL change	Model 1	0.98	0.75, 1.29	0.91	0.96	0.71, 1.31	0.79
	Model 2	0.93	0.71, 1.24	0.64	1.00	0.71, 1.41	0.95
	Model 3	0.95	0.71, 1.26	0.72	1.03	0.72, 1.47	0.88

Abbreviations: β, standardized effect estimate; CI, 95% confidence interval, BMI, body mass index; CI, confidence interval

Model 1: age + education + the time between audiometry and the PET scan.

Model 2: model 1 + APOE-4 carriership.

Model 3: model 2 + BMI + hypertension + diabetes + smoking + alcohol consumption.

Models for HL change are additionally adjusted for baseline HL.

### 3.4 Change in hearing loss and incident Aβ PET positivity

Similarly, we found no significant association between annual rate of change in HL and *incident* Aβ PET (Table 2**;** Fig. 3B). Furthermore, the relationship between the rate of change in HL and SUVR was also not significant (**Table S2; Fig. S2A**). There was also no significant association between the change in HL and the SUVR in the left and right superior temporal gyri (**Table S2; Fig. S2B-C**). For all aforementioned results, the findings were similar for the total population and the subsample without p-tau217 positive participants. Additionally, APOE4 did not modify the relationship with the mean SUVR, as evidenced by an insignificant and APOE4-by-HL interaction (β=0.13 [-0.10, 0.37], *p*=0.28; **Fig. S2D**).

### 3.5 Plasma AD biomarkers at baseline and subsequent hearing loss

**Fig. 4:**
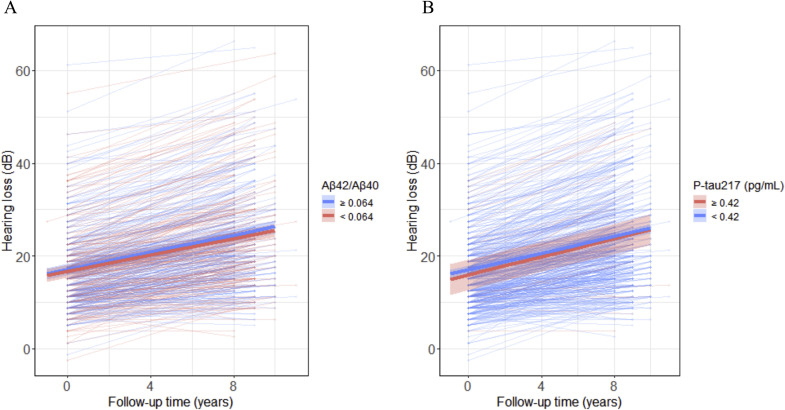
Trajectories of HL are plotted over time and for different baseline biomarker concentrations of **A** plasma Aβ42/Aβ40 and **B** plasma pTau217. Follow-up time (years) is depicted on the x-axis. HL is presented on the y-axis. Predicted trajectories are plotted for low versus high plasma biomarker concentrations (median split for Aβ42/Aβ40 and ≥ 0.42pg/mL for p-tau217). Lower Aβ42/Aβ40 ratio and higher p-tau217 indicate abnormality.

At baseline, neither p-tau217 nor Aβ42/Aβ40 levels were significantly associated with HL (p-tau217: model 1: β=-0.01 [-0.09, 0.08], *p*=0.86; model 2-3: *p*>0.05; Aβ42/Aβ40: Model 1: β=0.03 [-0.05, 0.11], *p*=0.42; Model 2-3: *p*>0.05, Fig. 4).

In addition, the interaction between baseline p-tau217 and time was not significant, indicating that p-tau217 levels at baseline did not predict changes in HL over time (model 1: β=0.00 [-0.02, 0.03], *p*=0.72; model 2: β=0.00 [-0.02, 0.03], *p*=0.72; model 3: β=0.00 [-0.02, 0.03], *p*=0.72; Fig. 4A). Similarly, baseline Aβ42/Aβ40 levels did not predict HL trajectories (model 1: β=0.00 [-0.02, 0.03], *p*=0.91; model 2: β=0.00 [-0.02, 0.03], p=0.90; model 3: β=0.00 [-0.02, 0.03], *p*=0.91, Fig. 4B). When expressed in original units, each 0.1 pg/mL increase in baseline p-tau217 was associated with a 0.1 dB decrease in HL at baseline and a 0.01 dB annual increase in HL over time, however, neither the cross-sectional nor longitudinal association were significant. These findings indicate that the rate of HL did not vary by baseline biomarker levels (**Table S3**).

## 4 Discussion

In this longitudinal study, we found no bidirectional association between hearing loss (HL) and AD pathology. Baseline HL did not predict longitudinal changes in plasma p-tau217 levels nor incident Aβ PET positivity at follow-up. The change in HL over time was also unrelated to Aβ PET positivity. Analyses restricted to the superior temporal gyrus, which encompasses the primary auditory cortex and has been proposed as a site of HL origin [40,[45], [46], [47]]^,^ revealed no association between HL and Aβ pathology. This suggests that Aβ PET pathology is also not specifically elevated in auditory-related regions among subjects with more severe HL. Furthermore, APOE4 carriership did not modify these relationships. Similarly, baseline p-tau217 and Aβ42/Aβ40 levels were not predictive of longitudinal changes in HL.

The null associations between HL and Aβ PET positivity are consistent with prior studies [15,[17], [18], [19]]_._ While some studies have reported associations, they are limited by several constraints. These include small sample sizes [14,16,23,24], mainly focus on slight HL impairment [23,24], and the absence of healthy control participants [16]. In addition, the majority of these studies failed to consider APOE4 carriership [14,16,23,24]. The established association between APOE4 and Aβ PET positivity suggests the possibility of confounding effects by genetic risk in the previous studies [48,49]. Previous studies on the association between HL and APOE4 carriership are inconclusive. A study reported lower HL in APOE4 carriers, while another study found no significant relationship [50]. Besides controlling for APOE4 carriership, we also investigated a potential effect modification by APOE4 carriership. We hypothesized that associations between HL and Aβ pathology might be stronger in APOE4 carriers. This was motivated by a recent study reporting a stronger association between HL and dementia risk in APOE4 carriers compared to non-carriers [28]. Adjusting for APOE4 carriership or its interaction with HL did not alter our results, but the absence of APOE4 corrections in prior studies remains a key limitation. Findings may be incorrectly attributed to HL if APOE4 carriership is unequally divided across control and HL participants.

In addition to PET imaging, we examined the relationship between HL and AD pathology using AD plasma biomarkers, including p-tau217 and Aβ42/Aβ40. Neither biomarker showed a significant relationship with baseline HL or the progression of HL over time. To the best of our knowledge, the present study is among the first to investigate the longitudinal association between objective HL measures and validated AD plasma biomarkers in a large population of cognitively unimpaired participants. The null findings regarding plasma biomarkers align with previous studies examining CSF Aβ42 and p-tau181 [12,[20], [21], [22],^51^,^52^]. However, this may reflect the timing of when the HL begins to influence the hearing function during disease progression. Two studies including participants with cognitive impairment have reported significant associations between HL and CSF p-tau181 as well as tau PET, suggesting that such relationships may become detectable only after substantial AD pathology has accumulated or when cognitive symptoms manifest [16,51].

We also examined the reverse relationship: whether HL predicted subsequent increases in plasma p-tau217 over time. Again, we found no evidence that HL accelerated p-tau217 accumulation. We hypothesized such an association based on animal studies reporting that mice with AD pathology exhibited greater HL [26,27]. Nevertheless, these animal models may not fully capture the mechanisms relevant to humans. Aging is typically induced in animal studies, which may not accurately reflect the natural aging process [53]. Moreover, the pathological processes of AD in mice may not be entirely analogous to those observed in humans, and findings may not translate well from mice to humans [54]. Taken together, our findings argue against a meaningful relationship between HL and AD pathology, at least in cognitively unimpaired individuals. Neither early AD pathology nor HL appears to significantly influence the progression of the other in preclinical stages.

The Lancet Commissions (2024) suggested that HL is specifically related to AD [3,4]. Accumulation of Aβ in the brain is thought to be the primary influence driving AD pathogenesis [55]. However, our findings do not indicate a relationship between HL and Aβ deposition or AD plasma biomarkers. Therefore, the observed association between HL and AD may be attributable to other influences, such as a shared neurodegenerative pathology [56]. Furthermore, the increasing prevalence of HL and AD with advancing age suggests that residual aging effects, which may not have been fully accounted for, may drive associations reported in the literature.

Additionally, other pathways have been suggested in the literature that may explain the association between HL and dementia. The information degradation theory states that HL leads to an increased demand for cognitive resources, directing resources towards hearing and thereby reducing overall cognitive performance [[57], [58], [59]]. Moreover, in the sensory deprivation theory, it is hypothesized that HL affects cognition via pathological structural brain changes [4]. For example, in our recent work, HL was related to poorer white matter microstructure in auditory-related tracts HL [60]. Similarly, a study from the UK 1946 birth cohorts has shown that hearing loss at age 70 predicted faster rates of whole brain and hippocampal volume atrophy [61]. Our insignificant findings suggest that these alternative theories, if relevant, may result in pathological changes not specific to AD. Taken together, Aβ PET pathology and early-stage AD biomarkers seem not to be involved in the association between HL and AD dementia.

### 4.1 Strengths and limitations

A major strength of our study is the availability of longitudinal assessment of both HL and plasma p-tau217 levels, which allows for the examination of relationships over time. Additionally, we included complementary early AD biomarkers, namely p-tau217, a marker of soluble hyperphosphorylated tau, and Aβ PET, a marker of cortical beta-amyloid plaques. This enabled a comprehensive assessment of the association between AD pathology and HL. The present study employed a large sample size compared to previous studies on this topic, resulting in strong statistical power and reliable results, in combination with the large number of well-documented possible confounders. This study is noteworthy for its unique approach in investigating the hypothesized association between HL and AD pathology in both directions within the same population.

Several limitations should be noted. Although we had longitudinal measures of plasma p-tau217 levels, our study lacked true longitudinal measures of Aβ PET, which limited the assessment of causality on Aβ accumulation. Moreover, while the seven-year interval between baseline HL and Aβ PET was longer than most studies in the literature, it still captures only a short window in the protracted AD disease trajectory and may have constrained our ability to detect associations that emerge over extended periods. To strengthen directional inference in Aβ PET analyses, we excluded p-tau217 positive participants (and thus likely to be Aβ PET-positive) at baseline to assess incident Aβ PET positivity at follow-up. Sensitivity analyses indicated that excluding these participants did not alter the observed relationships, supporting the robustness of our findings. Additionally, the subgroup of participants who underwent PET imaging is healthier than the overall study population of the Rotterdam Study, partly because PET eligibility required the absence of dementia diagnosis. Therefore, we cannot exclude the possibility of an association between HL and AD pathology in individuals with dementia. This may have introduced a selection bias, with a potential underestimation of the actual strength of the relationship. The relatively mild hearing losses (average baseline HL of 17.2 dB) and low prevalence of hearing impairment (15.8% at baseline, including very few participants with severe HL), which are comparable to other population-based studies [[62], [63], [64]], may reflect associations commonly observed in the general population, but they do not exclude the potential relationship between more severe HL and AD pathology. We excluded participants with conductive hearing loss to eliminate any temporary conductive impairments. In doing so, we may have excluded some participants with chronic conductive hearing loss. However, the number of such cases is likely small and therefore unlikely to meaningfully affect the results [65]. Furthermore, the effects of HL on AD are thought to be most evident during midlife [3]. Our participants were late-midlife adults, with an average age of 62.37 years at baseline, which may have contributed to the lack of significant associations. Taken together, associations between HL and AD markers may be stronger in a younger population with larger hearing losses.

Overall, HL was not associated with either Aβ PET positivity or AD plasma biomarkers in the present study. However, it has been hypothesized that central auditory functioning may be an indicator of cognitive decline [66,67]. Therefore, future research should explore whether central auditory measures are more strongly related to AD pathology. Furthermore, other pathways, as mentioned previously, that are hypothesized to contribute to dementia should be further investigated to assess whether HL specifically relates to AD dementia or other dementia types.

### 4.2 Conclusions

In conclusion, we found no evidence that HL is associated with AD biomarkers in either direction. These results suggest that the hypothesized relationship between HL and clinical symptoms of AD is unlikely to be mediated by core AD pathology (amyloid-beta and tau). Future research should investigate alternative mechanisms, like a shared neurodegenerative pathology or the sensory deprivation theory, to elucidate the association of HL with AD.

## Funding

This project has received funding from the European Union’s Horizon 2020 research and innovation program (MSCA-IF-GF no.101032288 to Dr Neitzel), ZonMW Memorabel grant 733050817 (to Dr Vernooij), and Alzheimer’s Association research grant (AARG-22-972229 to Drs Vernooij and Neitzel), Erasmus MC fellowship (to Dr Neitzel), Alzheimer Nederland Cross-Border grant (WE.03-2024-24 to Dr Neitzel), ABOARD, which is a public-private partnership receiving funding from ZonMW (73305095007), and Health∼Holland, Topsector Life Sciences & Health (LSHM20106 to Dr Vernooij) as well as TAP-dementia, a ZonMW-funded project (10510032120003 to Dr Vernooij) in the context of the Dutch National Dementia Strategy, and from the Heinsius Houbolt foundation (to Dr Kremer).

## Consent statement

Written informed consent was obtained from all participants.

## Declaration of generative ai and ai-assisted technologies in the writing process

During the preparation of this work, the authors used ChatGPT in early drafts to improve the structure and readability of the article. After using this tool, the authors reviewed and edited the content as needed and take full responsibility for the content of the publication.

## Data availability

Rotterdam Study data can be obtained upon request. Request should be directed towards the management team of the Rotterdam Study (secretariat.epi@erasmusmc.nl), which has a protocol for approving data requests. Because of restrictions based on privacy regulations and informed consent of the participants, data cannot be made freely available in a public repository. The Rotterdam Study has been entered into the Netherlands National Trial Register (NTR; www.trialregister.nl) and into the WHO International Clinical Trials Registry Platform (ICTRP; www.who.int/ictrp/network/primary/en/) under shared catalogue number NTR6831.

## CRediT authorship contribution statement

**Jordi H.C. Boons:** Writing – review & editing, Writing – original draft, Methodology, Formal analysis, Conceptualization. **Phuong Thuy Nguyen Ho:** Writing – review & editing, Writing – original draft, Methodology, Formal analysis, Conceptualization. **Anna van Houwelingen:** Writing – review & editing. **M. Arfan Ikram:** Writing – review & editing. **Gertjan Dingemanse:** Writing – review & editing. **Bernd Kremer:** Writing – review & editing. **Meike W. Vernooij:** Writing – review & editing, Conceptualization. **Andre Goedegebure:** Writing – review & editing, Supervision, Methodology, Conceptualization. **Julia Neitzel:** Writing – review & editing, Supervision, Funding acquisition, Conceptualization.

## Declaration of competing interest

All authors declare that they have no known competing financial interests or personal relationships that could have appeared to influence the work reported in this paper.

## Appendix Supplementary materials

Image, application 1
