# Supplementary material for: No longitudinal association between hearing loss and Alzheimer’s disease pathology
Source: J Prev Alzheimers Dis. 2026 Jan 20;13(3):100481. doi: 10.1016/j.tjpad.2026.100481 (PMC12856869; doi:10.1016/j.tjpad.2026.100481)
Supplement: Supplementary file 1 [file mmc1.docx]

**APPENDIX**


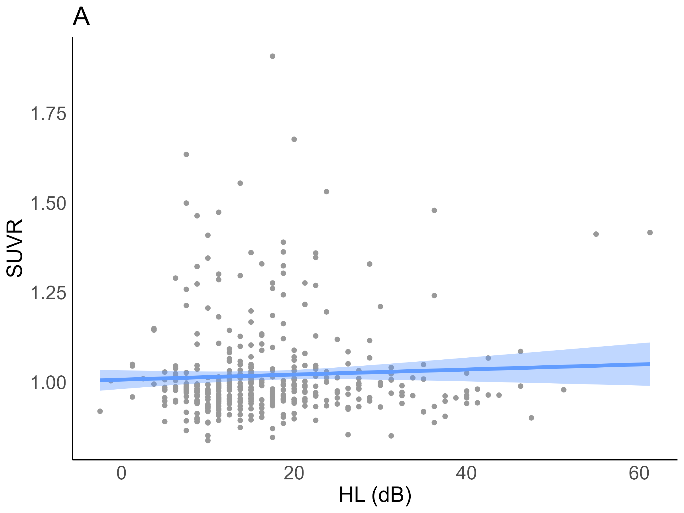

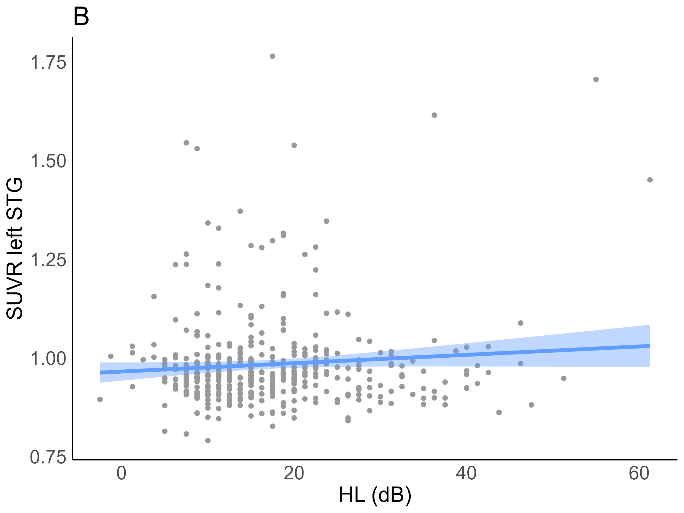

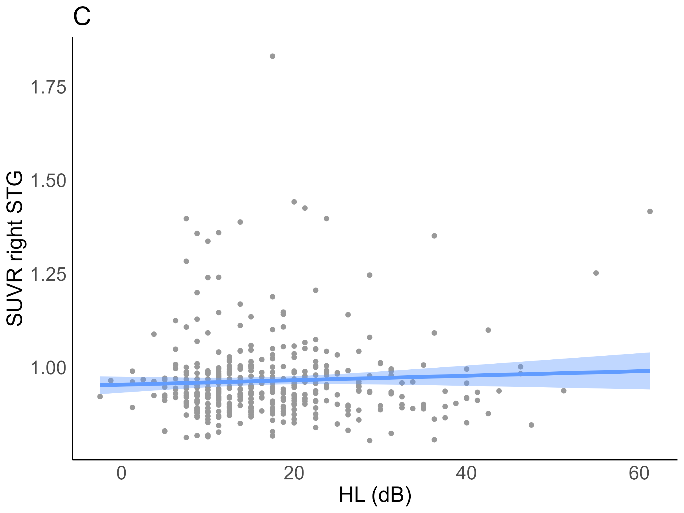

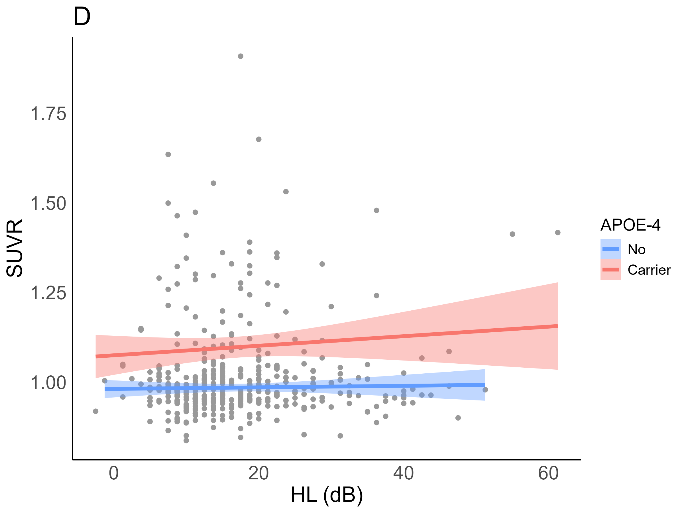


**Fig. S1.** Baseline HL plotted against mean SUVR values (**A)**, SUVR in the left STG (**B**), SUVR in the right STG (**C**). Panel (**D**) displays the mean SUVR again, while showing the interaction between HL and APOE4 carriership.


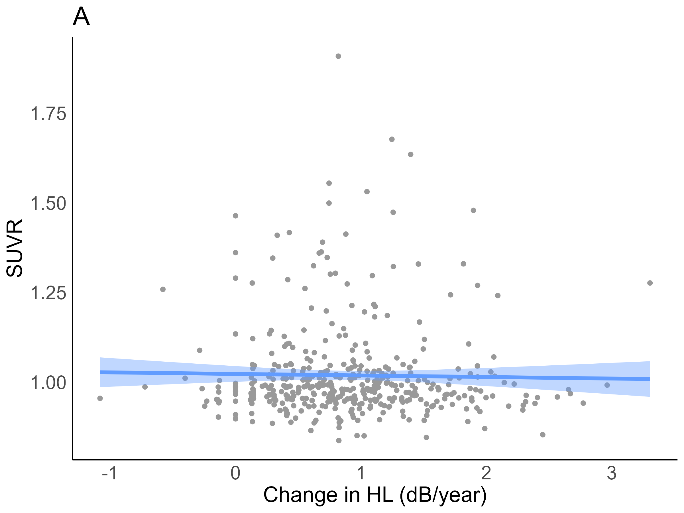

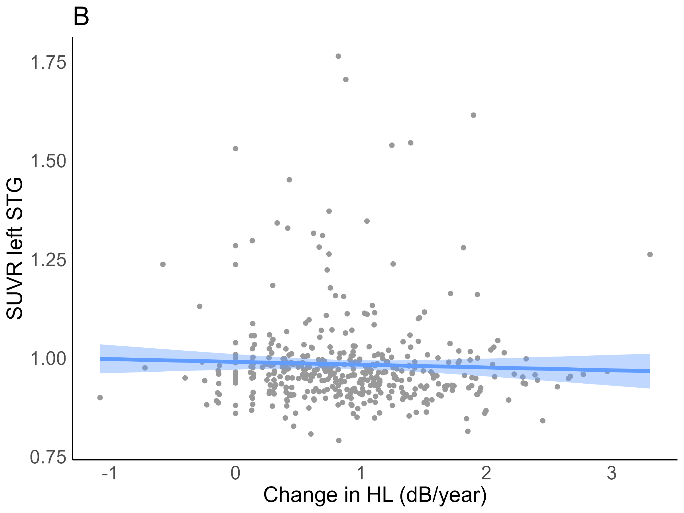

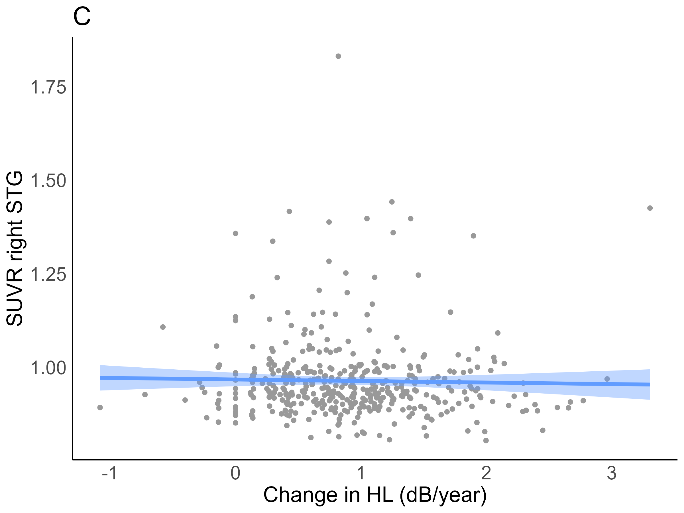

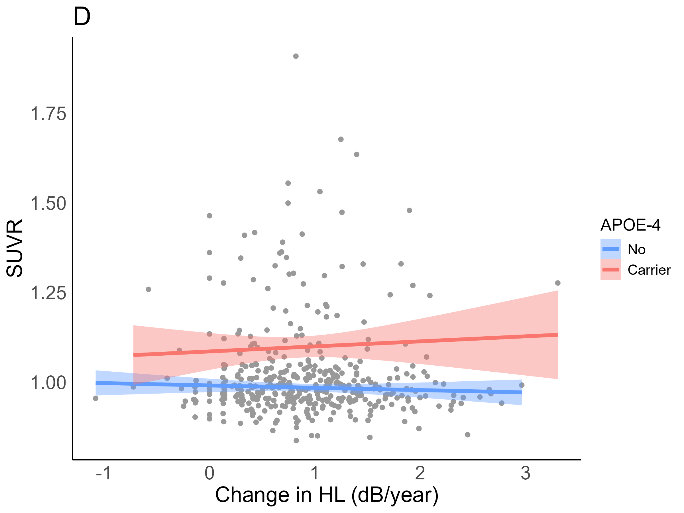


**Fig. S2.** Change in HL over time plotted against overall SUVR values (**A)**, SUVR in the left STG (**B**), SUVR in the right STG (**C**). Panel (**D**) shows the mean SUVR again, while showing the interaction between HL and APOE4 carriership.

**Table S1**

Associations between baseline hearing loss and subsequent plasma p-tau217 levels

| **Measure** |  | **β** | **95%CI** | ***p*** |
| --- | --- | --- | --- | --- |
| P-tau217 | Model 1 | 0.00 | -0.08, 0.08 | 1.00 |
|  | Model 2 | 0.00 | -0.08, 0.08 | 0.98 |
|  | Model 3 | 0.00 | -0.07, 0.08 | 0.93 |
| P-tau217 x time | Model 1 | 0.02 | -0.01, 0.05 | 0.27 |
|  | Model 2 | 0.02 | -0.01, 0.05 | 0.27 |
|  | Model 3 | 0.02 | -0.01, 0.05 | 0.26 |

Abbreviations: β, standardized effect estimate; CI, 95% confidence interval, BMI, body mass index; CI, confidence interval; SUVR, standardized uptake value ratio.

Model 1: age + education + the time between audiometry and the PET scan.

Model 2: model 1 + APOE-4 carriership.

Model 3: model 2 + BMI + hypertension + diabetes + smoking + alcohol consumption.

**Table S2**

Linear regression results of the associations between baseline hearing loss or change in hearing loss and Aβ PET SUVR

| **Measure** |  | **β** | **95%CI** | ***p*** | | **β** | **95%CI** | ***p*** |
| --- | --- | --- | --- | --- | --- | --- | --- | --- |
| ***All participants (N = 474)*** | | | | | ***Baseline p-tau217-negative participants (n = 429)*** | | | |
| **Mean SUVR** | | | | | | | | |
| Baseline HL | Model 1 | -0.03 | -0.15, 0.08 | 0.557 | | 0.00 | -0.13, 0.13 | 0.961 |
|  | Model 2 | -0.04 | -0.14, 0.06 | 0.402 | | -0.02 | -0.14, 0.10 | 0.769 |
|  | Model 3 | -0.04 | -0.15, 0.06 | 0.383 | | -0.02 | -0.14, 0.09 | 0.693 |
| HL change | Model 1 | -0.04 | -0.13, 0.05 | 0.402 | | -0.05 | -0.14, 0.05 | 0.333 |
|  | Model 2 | -0.03 | -0.11, 0.06 | 0.545 | | -0.03 | -0.11, 0.05 | 0.422 |
|  | Model 3 | -0.03 | -0.11, 0.05 | 0.486 | | -0.03 | -0.12, 0.05 | 0.432 |
| **SUVR superior temporal gyrus left** | | | | | | | | |
| Baseline HL | Model 1 | 0.00 | -0.15, 0.15 | 0.961 | | 0.05 | -0.13, 0.24 | 0.571 |
|  | Model 2 | 0.00 | -0.14, 0.13 | 0.952 | | 0.04 | -0.13, 0.22 | 0.635 |
|  | Model 3 | 0.00 | -0.15, 0.13 | 0.940 | | 0.04 | -0.14, 0.21 | 0.687 |
| HL change | Model 1 | -0.05 | -0.15, 0.05 | 0.347 | | -0.06 | -0.16, 0.04 | 0.248 |
|  | Model 2 | -0.04 | -0.13, 0.06 | 0.442 | | -0.05 | -0.15, 0.04 | 0.294 |
|  | Model 3 | -0.04 | -0.13, 0.06 | 0.421 | | -0.04 | -0.14, 0.05 | 0.310 |
| **SUVR superior temporal gyrus right** | | | | | | | | |
| Baseline HL | Model 1 | -0.01 | -0.13, 0.12 | 0.934 | | 0.01 | -0.13, 0.16 | 0.864 |
|  | Model 2 | -0.01 | -0.12, 0.10 | 0.823 | | 0.00 | -0.13, 0.14 | 0.975 |
|  | Model 3 | -0.01 | -0.12, 0.10 | 0.858 | | 0.00 | -0.13, 0.14 | 0.992 |
| HL change | Model 1 | -0.04 | -0.15, 0.07 | 0.482 | | -0.04 | -0.16, 0.07 | 0.470 |
|  | Model 2 | -0.03 | -0.13, 0.07 | 0.593 | | -0.03 | -0.14, 0.08 | 0.542 |
|  | Model 3 | -0.03 | -0.13, 0.07 | 0.533 | | -0.03 | -0.14, 0.08 | 0.541 |

Abbreviations: β, standardized effect estimate; CI, 95% confidence interval, BMI, body mass index; CI, confidence interval; SUVR, standardized uptake value ratio.

Model 1: age + education + the time between audiometry and the PET scan.

Model 2: model 1 + APOE-4 carriership.

Model 3: model 2 + BMI + hypertension + diabetes + smoking + alcohol consumption.

Models for HL change are additionally adjusted for baseline HL.

**Table S3**

Associations between baseline plasma AD biomarkers and subsequent hearing loss

| **Measure** |  | **β** | **95%CI** | ***p*** |
| --- | --- | --- | --- | --- |
| P-tau217 | Model 1 | -0.01 | -0.09, 0.08 | 0.86 |
|  | Model 2 | -0.01 | -0.09, 0.08 | 0.97 |
|  | Model 3 | -0.01 | -0.09, 0.08 | 0.90 |
| P-tau217 x time | Model 1 | 0.00 | -0.02, 0.03 | 0.72 |
|  | Model 2 | 0.00 | -0.02, 0.03 | 0.72 |
|  | Model 3 | 0.00 | -0.02, 0.03 | 0.72 |
| Aβ42/Aβ40 | Model 1 | 0.03 | -0.05, 0.11 | 0.42 |
|  | Model 2 | 0.03 | -0.05, 0.12 | 0.41 |
|  | Model 3 | 0.04 | -0.04, 0.12 | 0.30 |
| Aβ42/Aβ40 x time | Model 1 | 0.00 | -0.02, 0.03 | 0.91 |
|  | Model 2 | 0.00 | -0.02, 0.03 | 0.90 |
|  | Model 3 | 0.00 | -0.02, 0.03 | 0.91 |

Abbreviations: β, standardized effect estimate; CI, 95% confidence interval, BMI, body mass index; CI, confidence interval; SUVR, standardized uptake value ratio.

Model 1: age + education + the time between audiometry and the PET scan.

Model 2: model 1 + APOE-4 carriership.

Model 3: model 2 + BMI + hypertension + diabetes + smoking + alcohol consumption.
